# Quantitative electroencephalogram and machine learning to predict expired sevoflurane concentration in infants

**DOI:** 10.1007/s10877-025-01301-2

**Published:** 2025-05-17

**Authors:** Rachit Kumar, Justin Skowno, Britta S. von Ungern-Sternberg, Andrew Davidson, Ting Xu, Jianmin Zhang, XingRong Song, Mazhong Zhang, Ping Zhao, Huacheng Liu, Yifei Jiang, Yunxia Zuo, Jurgen C. de Graaff, Laszlo Vutskits, Vanessa A. Olbrecht, Peter Szmuk, Allan F. Simpao, Fuchiang Rich Tsui, Jayant Nick Pratap, Asif Padiyath, Olivia Nelson, Charles D. Kurth, Ian Yuan, BRAIN Collaborative Investigators, Minal Menezes, Suzette Sheppard, David Sommerfield, ZhengZheng Gao, DongXu Lei, Jijian Zheng, Mengmeng Ding, Panpan Chen, Bin Du, Abhusani Bhuju, Camille van Hoorn, Emilie Roden, Jimmy W. Huh, Shih-Shan Lang, Paula Hu, Rita Saynhalath, Proshad Efune, Gijo Alex

**Affiliations:** 1 Genomics and Computational Biology Graduate Group at the Perelman School of Medicine at the University of Pennsylvania, Philadelphia, PA USA; 2 Department of Anaesthesia, The Children’s Hospital at Westmead, Sydney, Australia; 3 School of Child and Adolescent Health, The University of Sydney, Sydney, Australia; 4 Department of Anaesthesia and Pain Medicine, Perth Children’s Hospital, Perth, Australia; 5 Perioperative Care Program, Perioperative Medicine Team, Telethon Kids Institute, Perth, Australia; 6 Institute for Paediatric Perioperative Excellence, The University of Western Australia, Perth, Australia; 7 Division of Emergency Medicine, Anaesthesia and Pain Medicine, Medical School, The University of Western Australia, Perth, Australia; 8 Department of Anaesthesia, Royal Children’s Hospital Melbourne, Parkville, Australia; 9 Departments of Paediatrics, and Critical Care, University of Melbourne, Parkville, Australia; 10 Anaesthesia Research and Melbourne Children’s Trials Centre, Murdoch Children’s Research Institute, Melbourne, Australia; 11 President of Sichuan Provincial Jiaotong Hospital, The Affiliated Hospital of Sichuan Academy of Medical Science &, Sichuan Provincial People’s Hospital, Sichuan, China; 12 Department of Anesthesiology, Beijing Children’s Hospital, Capital Medical University, National for Children’s Health, Beijing, China; 13 Department of Anesthesiology. Guangzhou Women and Children’s Medical Center, Guangzhou, China; 14 Department of Anesthesiology, Shanghai Children’s Medical Center, Shanghai, China; 15 Shanghai Jiao Tong University School of Medicine, Shanghai, China; 16 Shanghai Children’s Medical Center Guizhou Hospital, Shanghai, China; 17 Department of Anesthesiology at the Shengjing Hospital of China Medical University, Shenyang, China; 18 Department of Anesthesiology and Perioperative Medicine, The Second Affiliated Hospital and Yuying Children’s Hospital of Wenzhou Medical University, Wenzhou, China; 19 Key Laboratory of Pediatric Anesthesiology, Ministry of Education, Wenzhou Medical University, Wenzhou, China; 20 Key Laboratory of Anesthesiology of Zhejiang Province, Wenzhou Medical University, Wenzhou, Zhejiang China; 21 Department of Anesthesiology and Critical Care Medicine, Johns Hopkins University School of Medicine, Baltimore, MD USA; 22 Department of Anesthesiology, West China Hospital, Sichuan University, Chengdu, Sichuan China; 23 Department of Anesthesiology, Adrz-Erasmus MC, Goes, The Netherlands; 24 Department of Anesthesiology, Weill Cornell Medicine, New York, NY USA; 25 Department of Anesthesiology, Pharmacology, Intensive Care, and Emergency Medicine, University Hospitals of Geneva, Geneva, Switzerland; 26 Geneva Neuroscience Center, University of Geneva, Geneva, Switzerland; 27 Department of Anesthesiology and Perioperative Medicine, Nemours Children’s Hospital, Wilmington, DE USA; 28 Sidney Kimmel Medical College, Thomas Jefferson University, Philadelphia, USA; 29 Department of Anesthesiology and Pain Management, Division of Anesthesiology, Children’s Health System of Texas, University of Texas Southwestern Medical Center, Dallas, TX USA; 30 Outcome Research Consortium, Cleveland, OH USA; 31 Department of Anesthesiology and Critical Care Medicine, The Children’s Hospital of Philadelphia and the Perelman School of Medicine, University of Pennsylvania, Philadelphia, PA USA; 32 Department of Biomedical and Health Informatics, The Children’s Hospital of Philadelphia, Philadelphia, PA USA; 33 Department of Anesthesiology and Critical Care Medicine and Neurology and Pediatrics, The Children’s Hospital of Philadelphia and the Perelman School of Medicine, University of Pennsylvania, Philadelphia, PA USA

**Keywords:** Machine learning, Infant electroencephalogram EEG, Pediatric EEG anesthesia, Quantitative EEG anesthesia, SHAP EEG analysis

## Abstract

Processed electroencephalography (EEG) indices used to guide anesthetic dosing in adults are not validated in young infants. Raw EEG can be processed mathematically, yielding quantitative EEG parameters (qEEG). We hypothesized that machine learning combined with qEEG can accurately classify expired sevoflurane concentrations in young infants. Knowledge from this may contribute to development of future infant-specific EEG algorithms. Frontal EEG collected from infants ≤ 3 months were time-matched as one-minute epochs to expired sevoflurane (eSevo). Fifteen qEEG parameters were extracted from each epoch and eight machine learning models combined the qEEG to classify each epoch into one of four eSevo levels (%): 0.1–1.0, 1.0–2.1, 2.1–2.9, and > 2.9. 64 epochs formed the p*ost hoc* SHAP dataset to determine the qEEG that contributed most to the model. The remaining epochs were randomly split 50 times into 80/20 training/testing sets. Accuracy and F1-score determined model performance. 42 infants provided 4574 epochs. The top classifiers K-nearest neighbors, default multi-layer perceptron, and support vector machine achieved 67.5–68.7% accuracy. Burst suppression ratio and entropy β were the top contributors to the models. Post hoc analysis performed without burst suppression ratio yielded similar prediction performance. In young infants, machine learning applied to qEEG predicted eSevo levels with moderate success. Burst suppression ratio, the most important contributor, represented an *efficient* EEG feature that encapsulated underlying EEG changes seen on other qEEG features. These results provided insight into EEG parameter selection and optimal machine learning models used for future development of infant-specific EEG algorithms.

**Supplementary Information:**

The online version contains supplementary material available at 10.1007/s10877-025-01301-2.

## Introduction

Dosing of volatile anesthetics is traditionally based on minimal alveolar concentration (MAC) of a healthy age-matched population [1]. MAC, derived from patient movement during surgical incision may be more influenced by spinal cord and subcortical effect rather than cortical effect. As anesthesia consists of hypnosis, anti-nociception, and immobility, dosing based on MAC derived from a healthy population is likely simplistic and insufficient for many of the medically complex patients presenting for surgery today.

Recently, electroencephalography (EEG) that directly assesses cortical electrical activity has gained traction to assess anesthetic level, leading some panels to recommend EEG as a standard monitor to guide anesthetic dosing to the individual patient [2, 3]. Processed EEG monitors and proprietary indices (e.g., Narcotrend, PSI, BIS) were originally designed to facilitate EEG-guided anesthesia by eliminating the requirement for interpreting raw EEG waveforms, but guiding anesthesia based solely on the index also has several limitations [4, 5]. Furthermore, although proprietary EEG indices generally correlate with depth of anesthesia in adults and children, this is not true for infants (under one year old) [6, 7]. Myelination and synaptogenesis are incomplete at birth and evolve throughout infancy, resulting in EEG amplitude and frequency changes that were not taken into account during the development of proprietary EEG indices for adults and older children [6, 7].

The absence of a reliable EEG index to evaluate anesthesia depth in infants is significant. Infants are at greater risk for anesthetic complications compared to older children, such as hypotension from excess anesthesia [8, 9]. Furthermore, traditional anesthetic dosing based on MAC, with its assumption that younger children needed higher doses to prevent movement, may lead to excessive anesthesia in the brain. A recent multi-center study showed that 59% of infants under 3-months-old experienced isoelectric EEG during surgery, an electrically quiescentcortex associated with higher anesthetic dose and hypotension [10].

Raw EEG can be computationally processed with mathematical algorithms that summarize EEG characteristics over a time epoch, yielding objective and replicable quantitative EEG (qEEG) parameters. Commonly used processed EEG monitors (e.g. BIS) combines several qEEG parameters to derive its final proprietary EEG index [11]. This present exploratory study uses a combination of qEEG and machine learning (ML) to determine if there are other EEG parameters that can differentiate anesthetic dose in infants ≤ 3-months-old. Machine learning allows for the integration of multiple parameters to perform a task (e.g., classification) without *a-priori* knowledge of how those parameters relate or contribute to the task and can be useful to describe non-linear relationship. Results from this study will hopefully identify the qEEG parameters to focus on for future development of infant-specific EEG algorithms used to optimize dosing in this young population.

## Methods

### Patient population

This study re-analyzed EEG and eSevo collected on infants ≤ 3-months-old from a previous 15-center study [10]. The original study included children ≤ 3 years old undergoing sevoflurane or propofol anesthesia, postmenstrual age ≥ 36 weeks on day of surgery, and airway management with an endotracheal tube or a supraglottic airway. Exclusion criteria for the original study were: American Society of Anesthesiologists Physical Status (ASA-PS) > 3; brain malformations; recent pre-operative use of propofol infusion or ketamine; history of abnormal EEG or severe neurologic abnormalities; emergency surgery or surgery of the head, heart, or brain. The Institutional Review Board of the Children’s Hospital of Philadelphia, the data coordinating center of the original study, exempted the present study from full review.

### EEG data acquisition

EEG were obtained with Masimo Sedline (Irvine, CA USA) recorded from the forehead corresponding to four channels: Fp1-aFz, Fp2-aFz, F7-aFz, and F8-aFz. EEG signals were recorded at 178 Hz, 30 mm/sec, amplitude setting at 10uv/mm, and monitored to maintain impedance less than 14 kΩ. The research coordinators at each site started recordings at 10uv/mm and adjusted accordingly to maintain waveform visibility. EEG files, stored in *European Data Format*, were housed at the Children’s Hospital of Philadelphia.

### Expired sevoflurane analysis

eSevo concentrations were extracted from the electronic record, where eSevo was recorded at least once a minute. eSevo were categorized into four levels: A: 0.1–1.0%, B: 1.0–2.1%, C: 2.1–2.9%, and D: > 2.9%, roughly corresponding to emergence, light, deep, and very deep anesthesia, respectively, for this age group [10, 12]. The eSevo ranges for the four levels were selected based on Koch et al. findings of eSevo 1.7–2.1% for “light” anesthesia and 2.2–2.8% for “deep” anesthesia [12], in addition to our study showing that the mean eSevo during anesthetic maintenance was 2.3–2.4% [10].

A classification rather than regression prediction approach was taken, since during clinical dosing we target ranges instead of an exact number of sevoflurane concentration to achieve the desired depth of sedation/anesthesia. Furthermore, this young population has not previously been studied, and we opted for the simpler classification method in this exploratory study.

### EEG data analysis

EEG from intubation to emergence were extracted and time-matched as one-minute epochs to the eSevo concentrations at the end of the minute. Epochs with sevoflurane concentrations < 0.1% were removed and the rest were assigned to one of the eSevo levels described above. EEG epochs were assessed for peak and disconnect artifacts with function “remove_artefacts” default parameters from NEURAL, a MATLAB toolbox for EEG processing [13]. EEG artifacts were: (1) continuous zero values lasting > 1 s; (2) amplitude > 1500 μV; (3) consecutive values > 0.1 s; or (4) impulse-like changes with an amplitude difference > 200 μV. Epochs with ≥ 25% artifacts were excluded from further analysis.

Eligible epochs of EEG were processed with NEURAL by passing the EEG through a 30 Hz low-pass filter, followed by down-sampling to 64 Hz. Fifteen qEEG parameters were calculated for each epoch: relative spectral power (0–100%), connectivity coherence (0–1), and spectral entropy (0–1) across four frequency bands: δ (0.5–4 Hz), θ (4–7 Hz), α (7–13 Hz), and β (13–30 Hz), resulting in 12 parameters (3 × 4 bands), in addition to burst suppression ratio, spectral edge frequency (SEF) 50% and 90% [13, 14]. These 15 qEEG parameters were selected from 30 + NEURAL parameters, to represent both linear (e.g., spectral power ratio, spectral edge frequency) and non-linear (e.g., entropy and coherence) EEG changes [13]. Unless otherwise specified, the default NEURAL parameters were used to generate the fifteen qEEG parameters.

EEG can be described as oscillations at different frequencies. The EEG power–frequency relationship (power spectrum) was calculated using the “gen_spectrum” function with a Welch periodogram and a Hamming window of 2 s, resulting in a power spectrum with a frequency resolution of 0.5 Hz [13]. Relative spectral power using the “spectral_relative_power” function determined the percentage of EEG power within a given frequency band (e.g., θ band 4–7 Hz) compared to the EEG power over the entire frequency band (0.5–30 Hz). Connectivity coherence using function “connectivity_features” assessed the “synchrony” between left and right EEG channels, with “0” representing no synchrony and “1” complete synchrony [13, 15]. Coherence was calculated using Welch periodogram between channel pairs in the spectral domain. Each subject had four channels of EEG recorded, 2 lateral and 2 medial. The lateral channels (left and right) formed one coherence value. The medial channels (left and right) formed the second coherence value. In line with NEURAL’s defaults, the spectral and coherence features were averaged across channels (notably, the coherence features are normally computed by taking the median across channel-pairs, but this was equivalent to the mean for the two channel-pairs from four channels. Spectral entropy using the “spectral_entropy” function, treats the normalized power distribution in the frequency domain as a probability distribution and then calculates the Shannon entropy of that distribution. This essentially measures the “randomness” or “complexity” of the EEG within the frequency domain, with “0” being completely predictable and “1” completely random [13, 16]. Spectral entropy typically decreases with increased anesthetic depth, though differences exist across different sub-frequency bands [14, 17].

Coherence and entropy were separately calculated for each of the four frequency bands, instead of a singular value across frequences 0.5–30 Hz. This was designed to determine see if there were differences in coherence and entropy between each of the four frequency bands. For example, entropy β represents the amount of predictability in EEG signals in the β frequency band between 13 and 30 Hz, and in an earlier study was found to be best at distinguishing between different sleep states [14]. Burst suppression ratio quantified the percentage of suppression (i.e., “1” represents complete suppression over the one-minute epoch), and was derived in NEURAL by combining eight frequency and amplitude features into a support vector machine to classify burst *vs* suppressed portions of EEG [13, 18]. Spectral edge frequency 50% and 90% represented the EEG frequency with 50% and 90% of the EEG power below it. Generally, with increase sevoflurane dose, coherence, burst suppression ratio, and relative spectral power in lower frequency bands increases, whereas entropy in lower frequency bands, and spectral edge frequency 50% and 90% decreases. [6, 17, 19]

### Machine learning for expired sevoflurane classification

Using the Python *scikit-learn* package with default parameters in a multi-class classification, the qEEG parameters (features) for each one-minute epoch and corresponding expired sevoflurane levels were used to develop eight ML classification models: Logistic Regression (LR) Python function “LogisticRegression”, Decision Tree (DT) Python function “DecisionTreeClassifier”, Support Vector Machine (SVM) Python function “SVM”, K-Nearest Neighbors (KNN) Python function “KNeighborsClassifier”, Gaussian Naïve Bayes (GNB) Python function “GaussianNB”, Default Multi-Layer Perceptron (DMLP) with default Adam optimizer Python function “MLPClassifier”, Multi-Layer Perceptron (MLP) with stochastic gradient descent optimizer Python function “MLPClassifier”, and AdaBoost of a decision tree (ADA) Python function “AdaBoostClassifier” [20].

Logistic Regression models linear relationships and often serves as a baseline model for benchmarking ML, but can exhibit poor performance when features are correlated [21]. Decision Tree can perform internal feature selection and is interpretable; it learns to make “decisions” based on features but can be prone to overfitting [22]. Support Vector Machine uses higher-dimensional representations of features through kernel functions (radial basis function for this study), which allows it to model nonlinear relationships [23]. K-Nearest Neighbors relies on the similarity of samples to cluster and classify, with no assumption on how the data is related to the output, but can be sensitive to outliers with difficulty classifying rare cases [24]. Gaussian Naïve Bayes uses Bayes’ theorem and precomputed probabilities from the training data distribution to predict posterior probabilities of each class. GNB performs well on high-dimensionality data, but assumes that the features are independent and of equal importance; uncommon in most settings [25]. Default Multi-Layer Perceptron and Multi-Layer Perceptron are from the same class that operate by optimizing parameters that map features to different spaces, often in sequence, with nonlinear activation functions, thus enabling them to model nonlinear relationships. The default parameters in scikit-learn DMLP has one hidden layer of 100 neurons while the MLP has three hidden layers of 10, 5, and 2 neurons each. The default “relu” activation function was used. MLP can be prone to overfitting due to the large number of parameters relative to sample sizes [26]. AdaBoost of a Decision Tree operates as a meta-optimizer by iteratively fitting many copies of the model, while re-weighing incorrect classification on each iteration, until an ensemble of models is built that can classify the entire dataset. Due to its iterative weighing, ADA can be highly sensitive to data noise and outliers [27].

A subset of 64 samples randomly selected from the dataset were held back from algorithm training to calculate post hoc SHAP (SHapley Additive exPlanations) values. The remaining epochs were randomly split 50 times into training and testing sets (in 80/20 ratio) to permit 50 iterations of training and metric evaluation. After each iteration, the model generated from the training set was evaluated on the respective testing set. Performance metrics on the testing set included accuracy and F1-score. Accuracy assessed the overall correctness of predictions (correct / total predictions). F1-score assessed the harmonic mean of precision (positive predictive value) and recall (sensitivity); F1-score = 2 × (precision × recall)/(precision + recall) [28]. The F1-score gives equal importance to precision and recall, and the highest F1-score is 1 (perfect precision and recall). The F1-score can be used when there is an imbalance between the classes in the data.

In addition to each model’s overall prediction accuracy, the balanced accuracy and F1-score at each eSevo level were also calculated. (i.e., how well the model predicted that the epoch was level A *vs* not level A, for each of the four levels).

Post hoc SHAP analysis was performed on the 64 epochs that were excluded from training/testing to determine the qEEG parameters most important to the classification outcome, with the aim of improving interpretability of the ML outcomes. SHAP assesses the information gain for each feature in an additive manner to estimate the contribution of each feature to the prediction. A positive SHAP means that the feature "contributes" to a prediction (e.g., increase in the feature makes the prediction more likely), whereas a negative SHAP denotes the opposite. SHAP values are presented as absolute values, as this quantifies importance across both positive and negative contributions. The magnitude of the SHAP value is averaged over multiple iterations of the model, as individual iterations of the model can have slightly different SHAP values. Due to how *weights* are assigned in some models, a feature may have a SHAP of − 1 for one iteration but + 1 for another iteration but represent the same importance. The magnitude of a SHAP value should only be compared with the same model, due to possible differences in internal feature scaling by the models. For this study, the Python *SHAP* package was applied to the classification models with highest accuracy and SHAP values were averaged across the holdout subset of 64 samples for each model [29].

### Statistical analysis

Descriptive statistics were used to summarize patient demographics and classification metrics. Principal component analysis (PCA) were performed only on the qEEG parameters to assess the effect of data clustering from the same patients and sites, which could impact the distribution of qEEG parameters. The principal components were not used as input to the ML models.

## Results

**Fig. 1:**
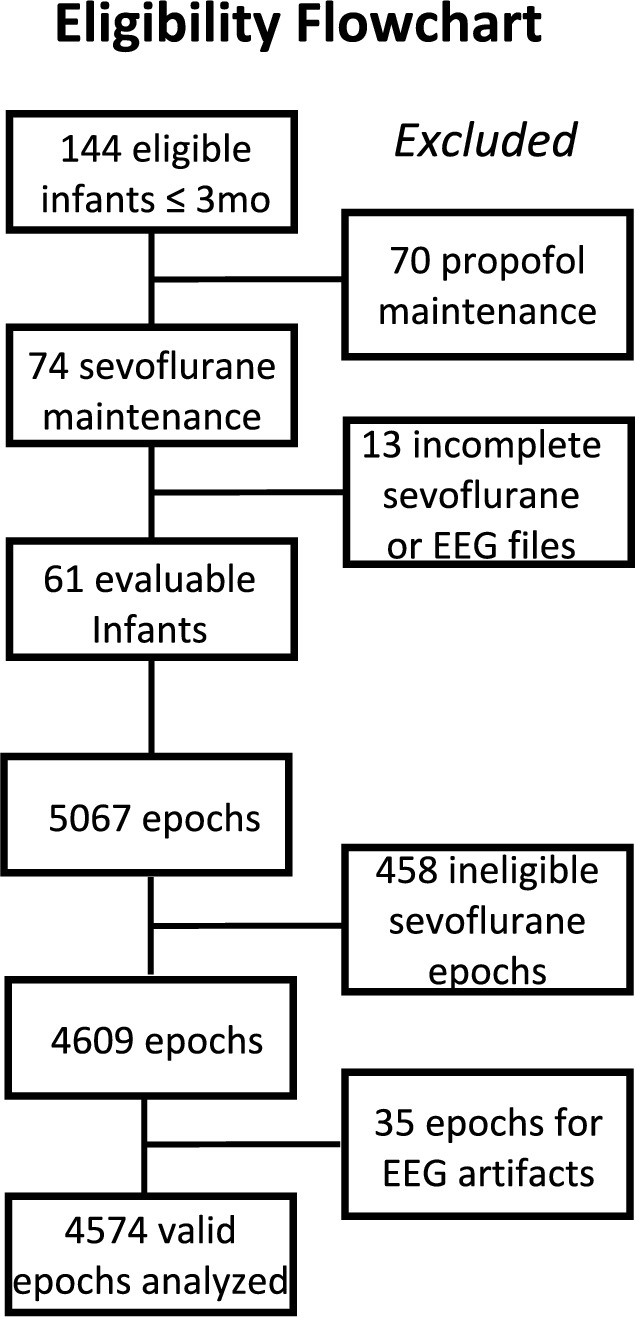
Eligibility flowchart

**Fig. 2:**
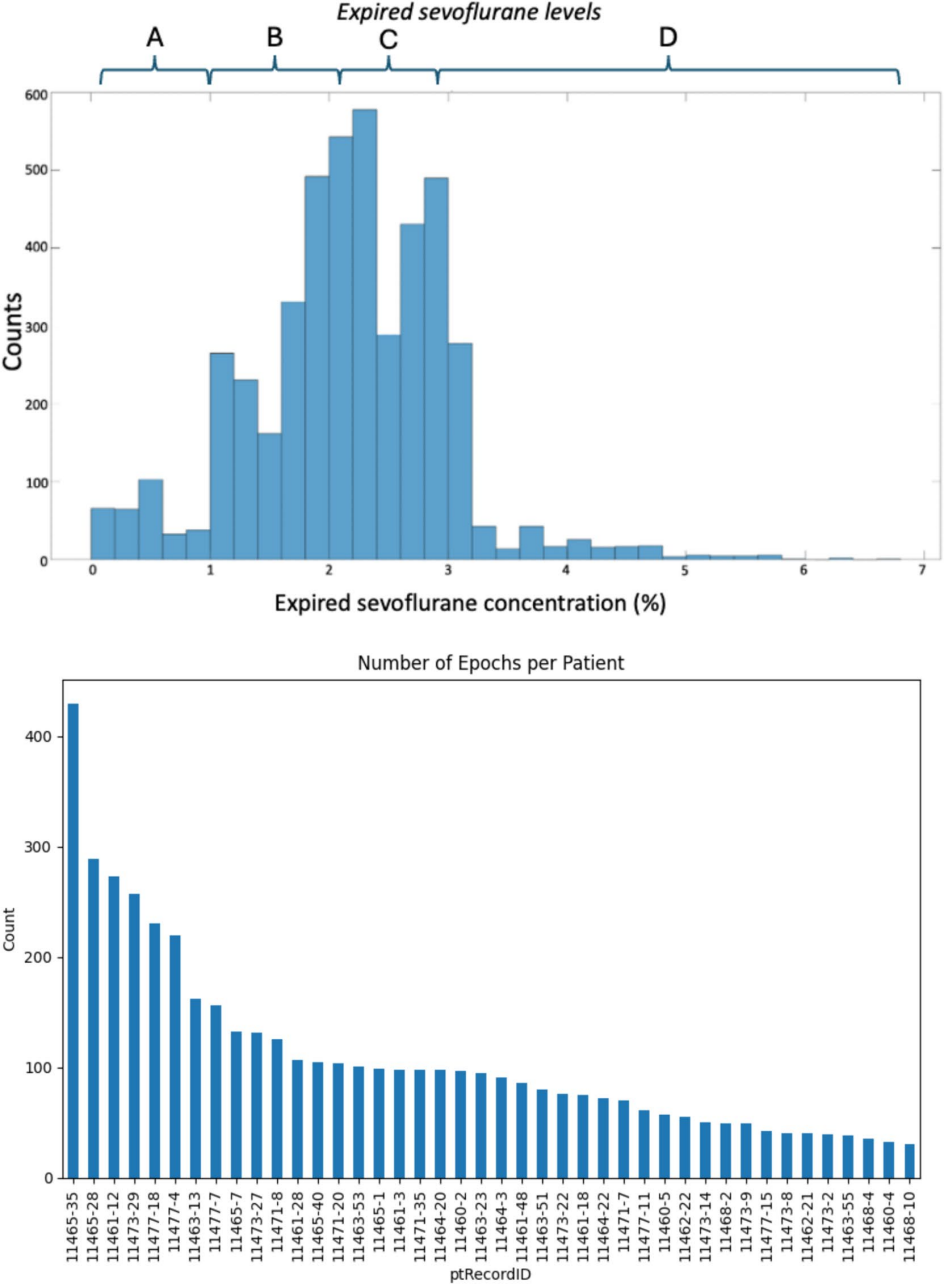
**A** Histogram of the distribution of analyzed expired sevoflurane concentrations. **B** Histogram of the number of epochs contributed by each subject

**Fig. 3:**
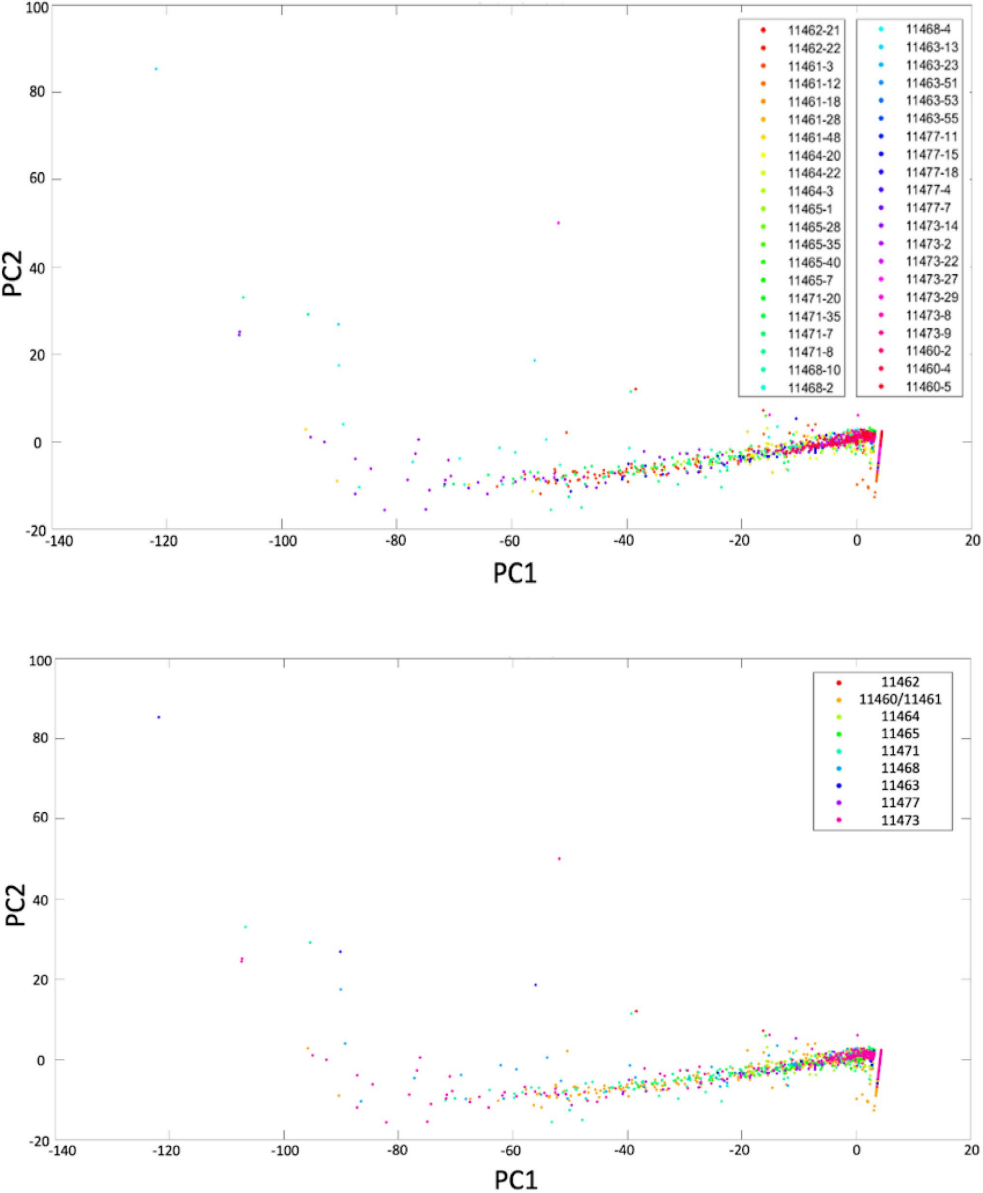
**A** Plot of the first two principal components of the quantitative EEG parameters colored by different infants, with little separation seen between the epochs from different infants. Each dot of the same color represents an epoch from the same infant. **B** Plot of the first two principal components of the quantitative EEG parameters colored by different sites, with little separation seen by site. Each dot of the same color represents an epoch from the same study site

Data were analyzed from sixty-one infants, producing 5067 one-minute EEG epochs and corresponding eSevo levels. From this, 4574 epochs from 42 unique infants were included in the final analysis (Fig. 1), after removing epochs where expired sevoflurane < 0.1% and EEG with artifacts > 25%. Table 1 shows the demographics and anesthetic management. 262, 1868, 1470, and 974 epochs in eSevo levels A, B, C, and D were analyzed, respectively (Fig. 2). Little to no clustering effects were identified on principal component analysis (Fig. 3), as both patients and sites were distributed consistently across the first two principal components. Table 2 shows the mean (stdev) for each qEEG at each of the four eSevo levels.

**Table 1:** Demographics and anesthetic management

N 42	Mean ± SD	Range
PMA day of surgery (weeks)	49.1 ± 4.2	(40.9, 56.1)
Weight (kg)	5.3 ± 1.3	(3.0, 9.1)

N 42	Count	%
Female	14	33
ASA PS		
1	12	29
2	24	57
3	6	14
Neuromuscular blockade for intubation	30	71
ETT	40	95
Propofol Bolus during induction	24	57

*PMA* post menstrual age, *SD* standard deviation, *ASA PS* American Society of Anesthesiology physical status, *ETT* endotracheal tube

**Table 2:** The mean and standard deviation of each of the 15 qEEG parameters for the four eSevo levels

	A		B		C		D	
	Mean	Stdev	Mean	Stdev	Mean	Stdev	Mean	Stdev
spectral_relative_power_delta	0.89	0.09	0.88	0.08	0.88	0.07	0.92	0.06
spectral_relative_power_theta	0.06	0.05	0.06	0.04	0.06	0.03	0.04	0.03
spectral_relative_power_alpha	0.03	0.03	0.04	0.03	0.04	0.03	0.02	0.02
spectral_relative_power_beta	0.02	0.02	0.02	0.02	0.02	0.01	0.01	0.01
connectivity_coh_delta	0.39	0.21	0.30	0.15	0.31	0.12	0.36	0.12
connectivity_coh_theta	0.23	0.23	0.13	0.13	0.12	0.12	0.12	0.11
connectivity_coh_alpha	0.26	0.22	0.17	0.13	0.18	0.10	0.20	0.10
connectivity_coh_beta	0.23	0.21	0.16	0.12	0.16	0.09	0.19	0.09
spectral_entropy_delta	0.69	0.16	0.74	0.08	0.74	0.09	0.70	0.09
spectral_entropy_theta	0.92	0.04	0.94	0.03	0.95	0.03	0.93	0.03
spectral_entropy_alpha	0.93	0.03	0.94	0.03	0.95	0.03	0.95	0.02
spectral_entropy_beta	0.94	0.03	0.93	0.04	0.90	0.03	0.93	0.03
SEF_50	0.67	0.29	0.76	0.33	0.78	0.29	0.65	0.26
SEF_90	2.98	1.86	3.79	2.31	3.91	2.04	2.69	1.44
burst_suppression	2.30	9.35	3.32	12.71	5.14	12.75	6.44	17.82

### Machine learning model performance

**Fig. 4:**
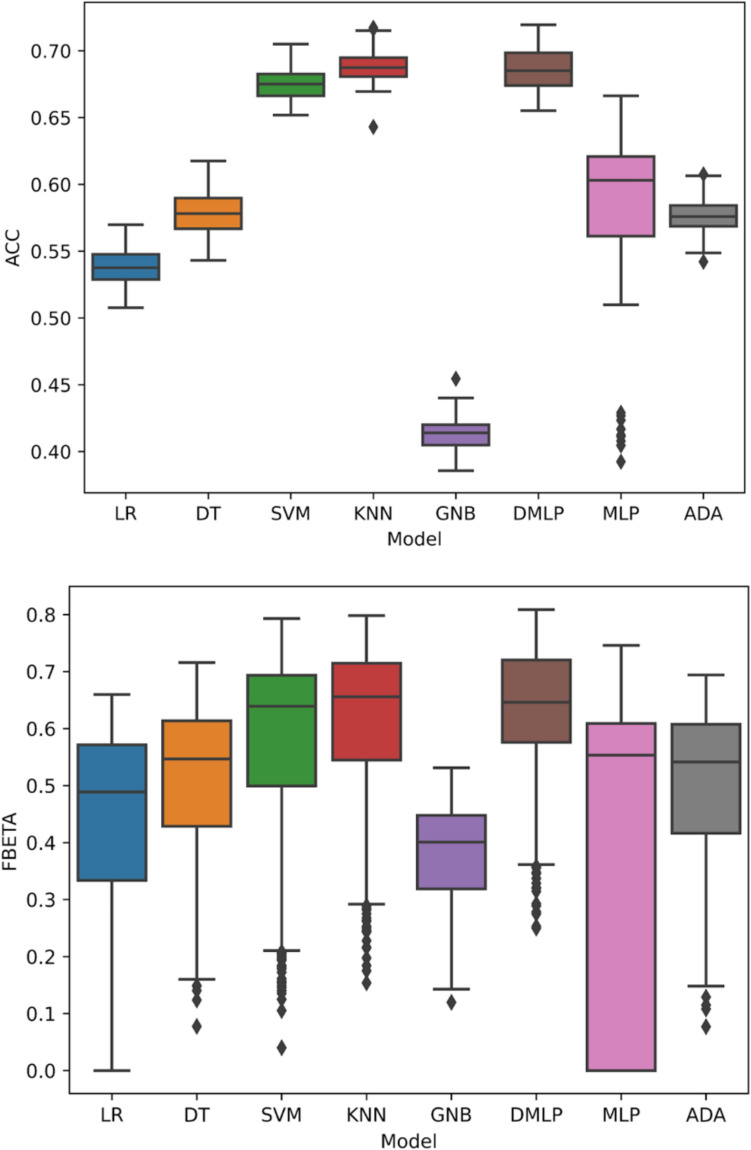
Boxplots of model overall accuracy (**A**) and F1-score (**B**) on holdout sequences over 50 different iterations. Center horizontal line represents median; top and bottom of box are 1st and 3rd quartile, respectively; top and bottom whiskers are 95th and 5th percentile, respectively; points outside of whiskers are outliers. *LR* logistic regression; *DT* decision tree, *SVM* support vector machine, *KNN* K-nearest neighbors, *GNB* Gaussian Naïve Bayes, *DMLP* default multi-layer perceptron (with default Adam optimizer), *MLP* multi-layer perceptron (with stochastic gradient descent optimizer), *ADA* AdaBoost of a decision tree

Figure 4 shows the performance of each classifier model. KNN, DMLP, and SVM were the top-performers with overall accuracies of median (interquartile range): 68.7% (68.1–69.5), 68.5% (67.4–69.8), and 67.5% (66.6–68.3), respectively. The top-performers for F1-score were also DMLP, KNN, and SVM, with median F1-scores all > 0.65. Gaussian Naïve Bayes had the worst overall accuracy 41.4% (40.5–42) and F1-score 40.1% (31.9–44.8).

Table 3 shows the prediction accuracy and F1-score at each of the four eSevo levels. For the two lowest levels (A, B), DMLP had the highest balanced accuracy and F1-score. For level C, KNN had the highest balanced accuracy and F1-score. For level D, DMLP had the highest balanced accuracy and KNN had the highest F1-score. The lowest eSevo level (A) had lower accuracy and F1-score compared to the higher levels (B-D).

**Table 3:** Balanced accuracy and F1-score stratified by expired sevoflurane concentrations (A: 0.1–1%; B: 1–2.1%; C: 2.1–2.9%; D: > 2.9%) for each model

Expired Sevoflurane levels	A: 0.1–1%		B: 1–2.1%		C: 2.1–2.9%		D: > 2.9%	
	Mean	Stdev	Mean	Stdev	Mean	Stdev	Mean	Stdev
Balanced accuracy								
LR	0.547	0.023	0.649	0.016	0.656	0.014	0.635	0.016
DT	0.595	0.035	0.721	0.013	0.675	0.017	0.700	0.018
SVM	0.561	0.018	0.786	0.012	0.743	0.011	0.749	0.016
KNN	0.605	0.026	0.800	0.013	**0.754**	**0.012**	0.766	0.017
GNB	0.586	0.023	0.589	0.010	0.628	0.016	0.639	0.017
DMLP	**0.640**	**0.029**	**0.802**	**0.013**	0.745	0.015	**0.773**	**0.017**
MLP	0.503	0.010	0.701	0.097	0.650	0.077	0.637	0.105
ADA	0.581	0.026	0.691	0.015	0.685	0.014	0.676	0.016
F1-score								
LR	0.165	0.070	0.615	0.020	0.532	0.020	0.423	0.029
DT	0.235	0.066	0.670	0.019	0.559	0.022	0.524	0.028
SVM	0.210	0.054	0.754	0.015	0.649	0.016	0.619	0.024
KNN	0.292	0.059	0.768	0.015	**0.666**	**0.018**	**0.640**	**0.024**
GNB	0.210	0.041	0.372	0.024	0.483	0.022	0.424	0.019
DMLP	**0.369**	**0.057**	**0.769**	**0.018**	0.653	0.021	0.640	0.026
MLP	0.009	0.035	0.690	0.054	0.465	0.228	0.354	0.261
ADA	0.237	0.061	0.647	0.020	0.574	0.020	0.495	0.027

*LR* logistic regression, *DT* decision tree, *SVM* support vector machine, *KNN* K-nearest neighbors, *GNB* Gaussian Naïve Bayes, *DMLP* default multi-layer perceptron (with default Adam optimizer), *MLP* multi-layer perceptron (with stochastic gradient descent optimizer), *ADA* AdaBoost of a decision tree

Bolded indicate best classifier for the expired sevoflurane level

### Post hoc SHAP analyses

**Fig. 5:**
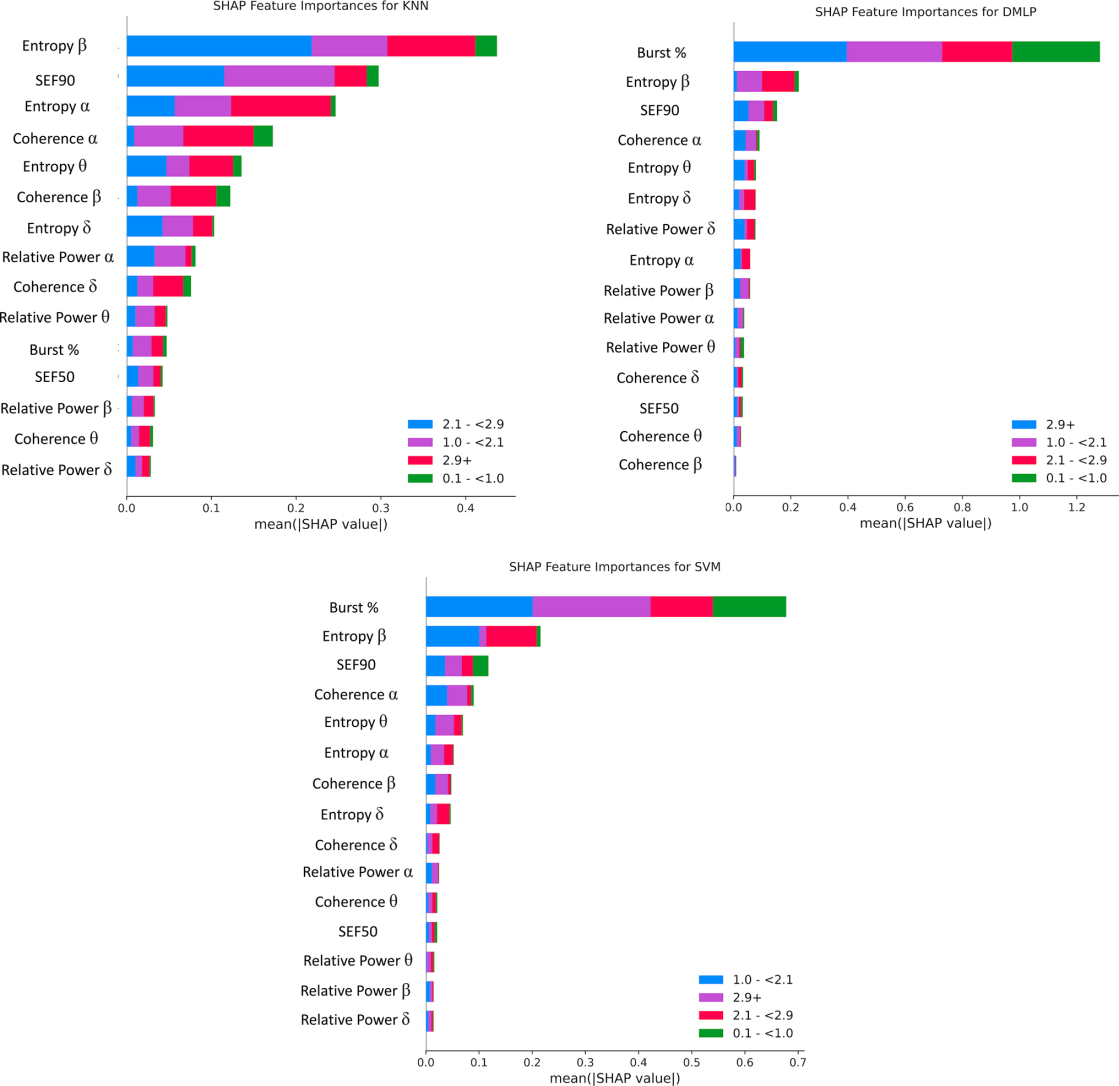
Plots of Shapley values for the three best-performing models (top = KNN, middle = DMLP, bottom = SVM). The four colors represent contribution to correctly classifying to one of the four levels of expired sevoflurane

**Fig. 6:**
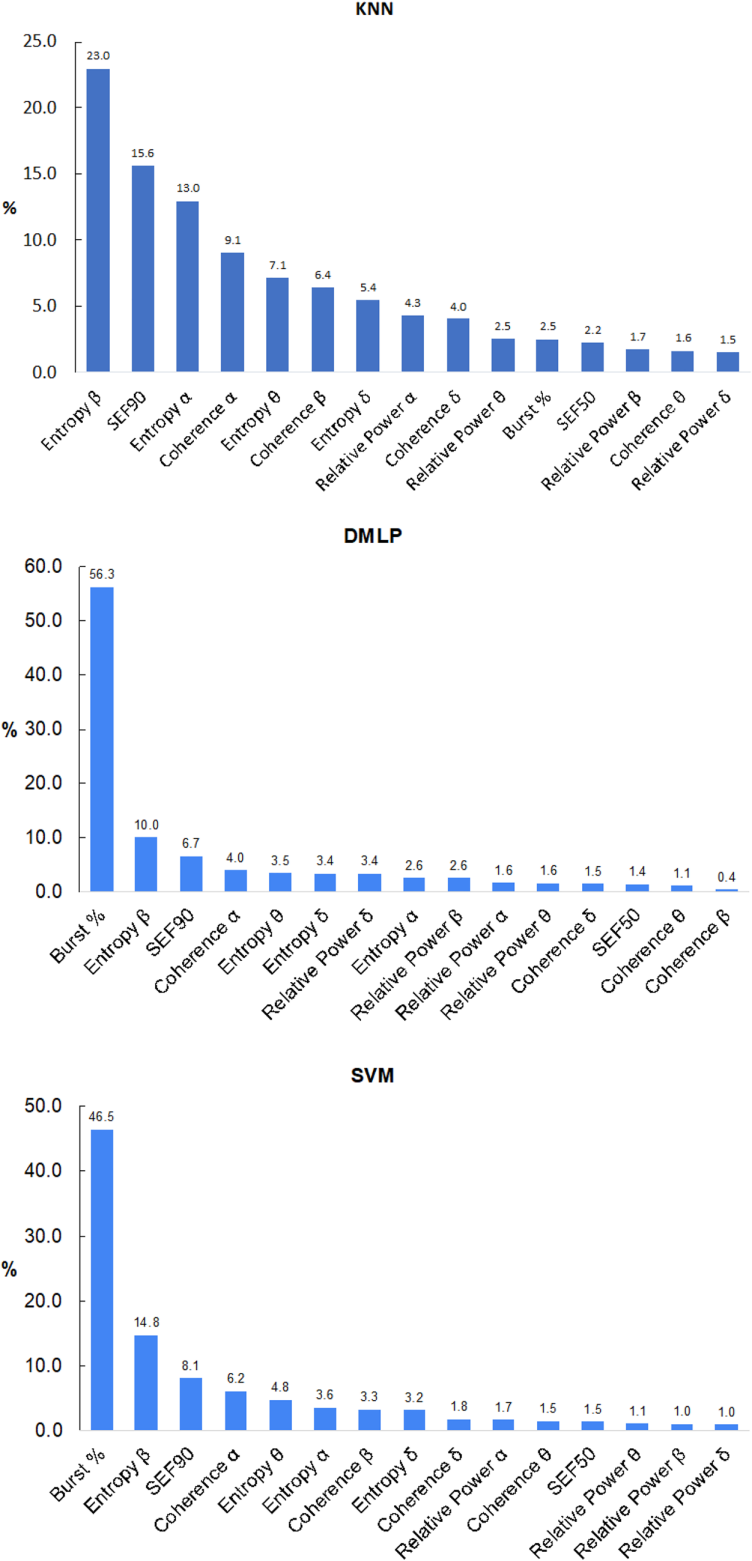
Ranked histogram of proportion (%) of each EEG features’ contribution to the model (top = KNN, middle = DMLP, bottom = SVM)

Figure 5 shows the mean SHAP values for the top-performers KNN, DMLP, and SVM, at each of the four eSevo levels. Figure 6 shows the percentage of each qEEG parameter’s contribution towards the SHAP values.

The most important qEEG features for SVM and DMLP were burst suppression ratio, entropy β, SEF90, coherence α, and entropy θ. Burst suppression ratio for eSevo levels A-D were 2.3, 3.3, 5.1, and 6.4%, respectively, and was the highest overall contributor to SVM and DMLP in classifying all four levels of eSevo. The other top model, KNN, showed a different distribution of parameter importance.

### Post hoc analyses without burst suppression ratio

Since burst suppression ratio accounted for a large proportion of the model’s behavior in SVM and DMLP, post hoc we excluded burst suppression ratio as a feature and re-analyzed the accuracy, F1-score, and SHAP values with the remaining 14 features.

Supplemental Fig. 1 shows the accuracy and F1-score without burst suppression ratio. The top performing models remained unchanged. The median accuracy decreased marginally for KNN: 68.7 to 68.5, DMLP: 68.5 to 68.1, and SVM: 67.5 to 67.2%. The median F1-score also decreased marginally for KNN: 65.6 to 65.0, DMLP: 64.6 to 64.2, and SVM: 63.9 to 63.5.

Without burst suppression ratio, supplemental Fig. 2 shows the SHAP values for KNN, DMLP, and SVM. Since burst suppression ratio contributed only 2.5% to the KNN model, the SHAP values remained unchanged after removing burst suppression ratio. Without burst suppression ratio in DMLP and SVM, the remaining top contributing features were the same, but the SHAP values decreased to levels similar to KNN (0.35–0.4). The percentage of each qEEG features’ contribution toward the SHAP values for KNN, DMLP, and SVM are displayed in supplemental Fig. 3. In DMLP and SVM without burst suppression ratio, the remaining features’ contribution increased proportionately to make up for the loss.

## Discussion

Processed EEG indices have been used extensively to guide anesthesia dosing in adults and older children, but valid EEG indices do not exist for the younger infant. This puts them at risk for unnecessarily deep anesthesia and hypotension among other complications [8, 9]. Despite concerns of “over-dosing” the infant brain, MAC have traditionally informed us that younger infants require higher anesthetic doses compared to older children [30]. These contradictions have prompted the search for EEG parameters to guide anesthesia in young infants. This study improves our understanding of the qEEG parameters and ML algorithms that can predict eSevo levels with moderate success in infants ≤ 3 months-old, with burst suppression ratio being the most important qEEG parameter.

In adults, SVM combined with two qEEG parameters observed a Cohen’s Kappa score of 0.80 with the BIS index during transitions of states of consciousness [31]. Another adult study used artificial neural network and four qEEG parameters to distinguish four levels of unconsciousness, with sensitivities of 86.4% (awake), 73.6% (light anesthesia), 84.4% (general anesthesia), and 14% (deep anesthesia) [32]. In both adult studies, BIS, a proprietary EEG index, was used as the “gold standard” for determining anesthetic depth. This study design cannot be applied to infants under 3 months-old, as these proprietary indices have not been validated in this younger group and are generally unreliable until closer to one year of age [33].

Although similar studies in infants under anesthesia do not exist, ML and qEEG were used in infants during natural sleep to distinguish states of consciousness [14, 34–38]. SVM combined with 57 qEEG parameters classified quiet and active sleep with 85% accuracy, 83% sensitivity, and 87% specificity [35]. Deep learning networks and hidden Markov models predicted quiet *versus* active sleep with an accuracy of 71–76% [36]. In term and pre-term neonates, two studies separately used ML to predict four stages of sleep with overall accuracies of 64% and 62%, respectively [37, 38]. The last two studies are closest to our study, in terms of patient population and results. Although unconsciousness during natural sleep may be different from general anesthesia, similar to the current study, entropy β decreased with increased sedation and could distinguish between different levels of consciousness, suggesting potential commonality [14].

SHAP analysis showed that burst suppression ratio was most significant in differentiating eSevo levels. In adults, burst suppression or deep anesthesia have been associated with increased postoperative delirum [39, 40]. The consequences of burst suppression are less clear in children. One study found that isoelectric EEG (low amplitude EEG for ≥ 2 secs) was common in young infants, and related to hypotension, higher eSevo, and lower Pediatric Quality of Life scores.

After excluding burst suppression ratio in post hoc analysis, the accuracy and F1-score did not decrease as expected, and the remaining EEG features’ contribution increased proportionately. This suggest that the remaining EEG features shared underlying characteristics with burst suppression ratio to make up for its absence (e.g., with increased anesthetic depth suppression ratio, SEF90, and alpha power ratios are all expected to decrease). Burst suppression ratio may represent an “efficient” qEEG that encapsulates the same underlying EEG changes seen on other more complicated EEG features in the infant population. Fortunately for clinicians, raw EEG waveforms are available on most commercial intraoperative EEG monitors and burst suppression and isoelectric EEG can be recognized and avoided with minimal training.

This study adds value and clinical relevance in two ways. We now understand what the most useful qEEG parameters are to differentiate eSevo levels in this young population (e.g., burst suppression ratio, entropy β, SEF90, etc.…). If an infant has qEEG parameters that correspond to a certain level of eSevo that doesn’t match the current level of eSevo, then we can use this knowledge to titrate the current eSevo dose. This often occurs in older children with neurodevelopment delays, where the required eSevo is often much lower than what would be predicted based on MAC [41]. In our practice, we advocate using burst suppression / isoelectric EEG to adjust dosing in neonates [42]. Future algorithms may further increase clinical relevance by finding the optimal “weight” to assign to the top qEEG parameters, with the goal of deriving a blended EEG index similar to the BIS, but specific for young infants.

This study has several limitations. Since no validated EEG index exist as the “gold standard” to differentiate anesthetic depth in this age group, we chose eSevo concentration as a practical proxy, while acknowledging that we are asking the inverse of the common question “what sevoflurane dose does this infant’s brain require for optimal anesthetic depth”. Due to the observational nature of the original EEG study, surgical stimulus and other intraoperative medications (e.g., propofol during induction, neuromuscular blockade) were not controlled for and could confound the EEG, though most of these medications were given prior to intubation. Our findings of lower prediction accuracy compared to adults may be due to (1) infants having limited amplitude and frequency ranges compared to adults, resulting in less discriminatory power between eSevo levels; (2) limited EEG data corresponding to the lowest eSevo level; and (3) lack of a “gold standard” EEG index as mentioned above. The data set is split on epochs rather than patients during training, meaning that the training models may have learned individual specific features that may lead to over-fitting the test set. However, principal component analysis showed little to no separation by either patient or site, indicating that the feature distributions in our training and validation sets are nondiscriminatory for individuals and sites. The down-sampling of EEG to 64 Hz may raise concern about potential aliasing. Lastly, during states of burst suppression (2.3–6.4% of the time), qEEG parameters such as relative spectral power, may be concentrated to the lower frequency bands and are less sensitive in distinguishing different eSevo levels. qEEG parameters, particularly ones that utilize frequency domain calculations, are reliant on EEG amplitude and frequency and can be very low during these low-amplitude states.

## Conclusion

In infants ≤ 3 months-old, quantitative EEG combined with machine learning models provided close to 70% accuracy in predicting eSevo levels. Burst suppression ratio was the most important contributor to the prediction model, representing an “efficient” EEG feature that encapsulated underlying EEG changes seen on other qEEG features. Results from this study provided insight into qEEG parameter selection and optimal ML models for future development of infant-specific EEG monitoring.

## Supplementary Information

Below is the link to the electronic supplementary material.

Supplementary file1 (DOCX 11126 kb)

## Data Availability

No datasets were generated or analysed during the current study.
