# Supplementary material for: Quantitative electroencephalogram and machine learning to predict expired sevoflurane concentration in infants
Source: J Clin Monit Comput. 2025 May 17;39(5):999–1014. doi: 10.1007/s10877-025-01301-2 (PMC12474631; doi:10.1007/s10877-025-01301-2)
Supplement: Supplementary file 1 — Supplementary file1 (DOCX 11126 kb) [file 10877_2025_1301_MOESM1_ESM.pdf]

Supplemental Figure 1: Without burst suppression ratio, boxplots of model overall accuracy (A) and F1-score on holdout sequences over 50 different iterations. Center horizontal line represents median; top and bottom of box are 1st and 3rd quartile, respectively; top and bottom whiskers are 95th and 5th percentile, respectively; points outside of whiskers are outliers.

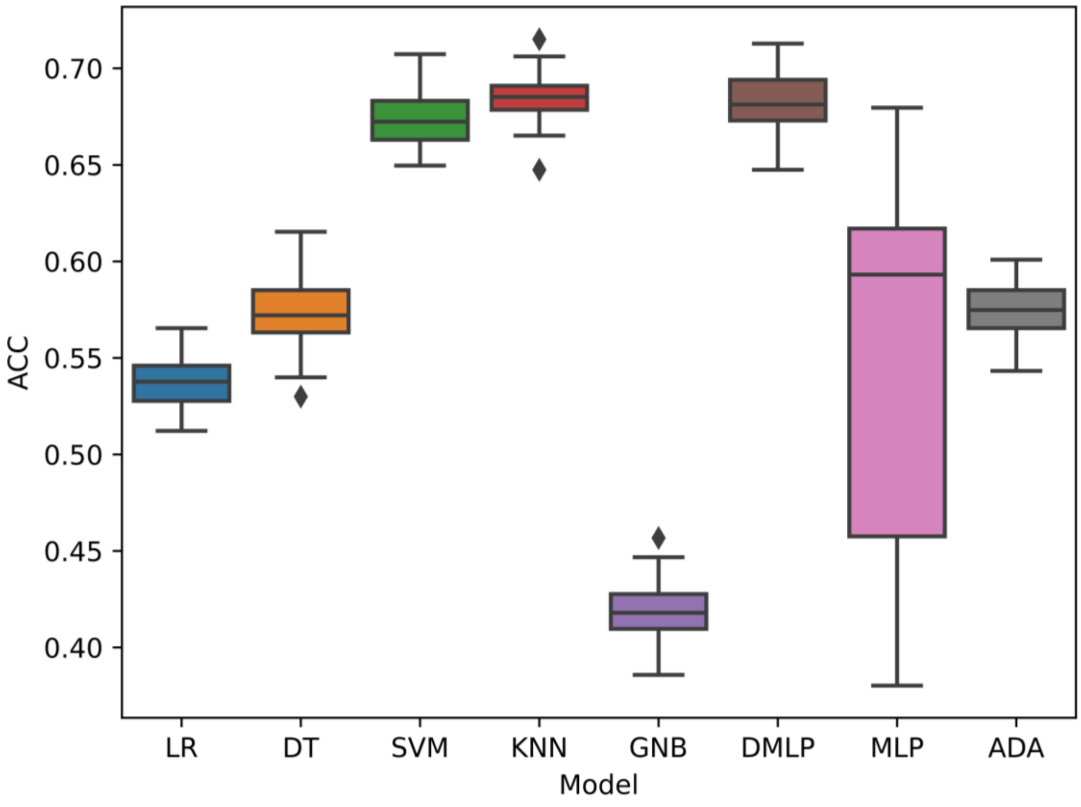

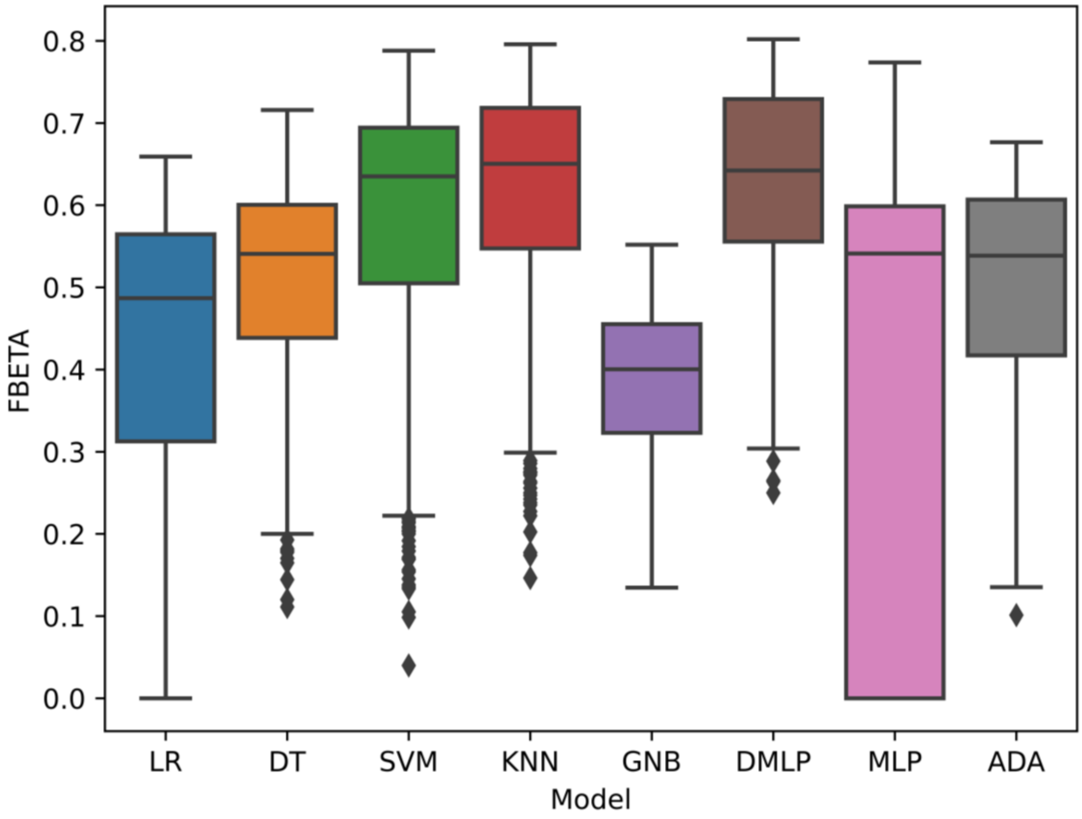

Supplemental Figure 2: Without burst suppression ratio, plots of Shapley values for the three best-performing models (top = KNN, middle = DMLP, bottom = SVM). The four colors represent contribution to correctly classifying to one of the four levels of expired sevoflurane.

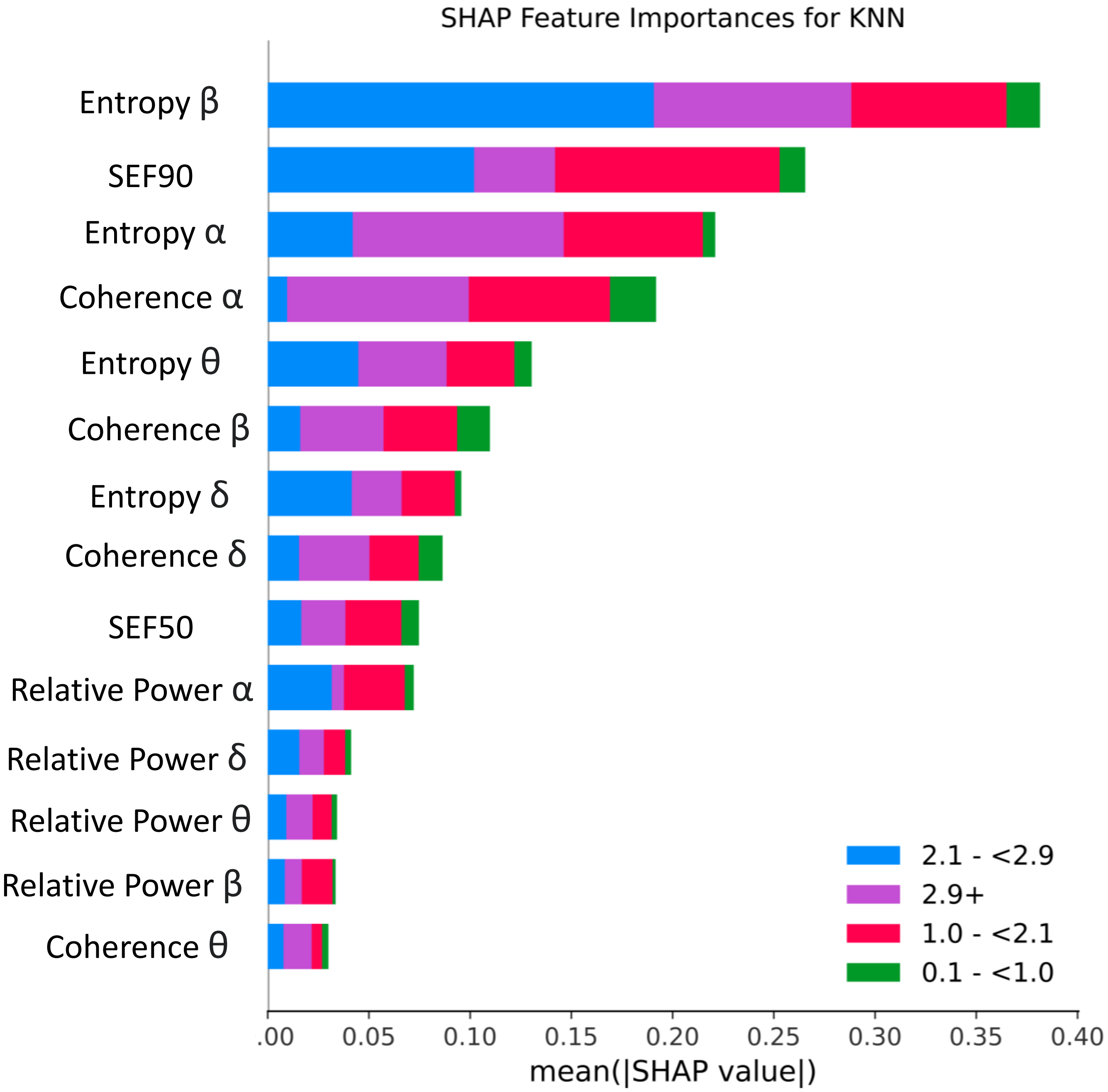

SHAP Feature Importances for KNN
Entropy β
SEF90
Entropy α
Coherence α
Entropy θ
Coherence β
Entropy δ
Coherence δ
SEF50
Relative Power α
Relative Power δ
Relative Power θ
Relative Power β
Coherence θ
2.1 - <2.9
2.9+
1.0 - <2.1
0.1 - <1.0
.00
0.05
0.10
0.15
0.20
0.25
0.30
0.35
0.40
mean(|SHAP value|)

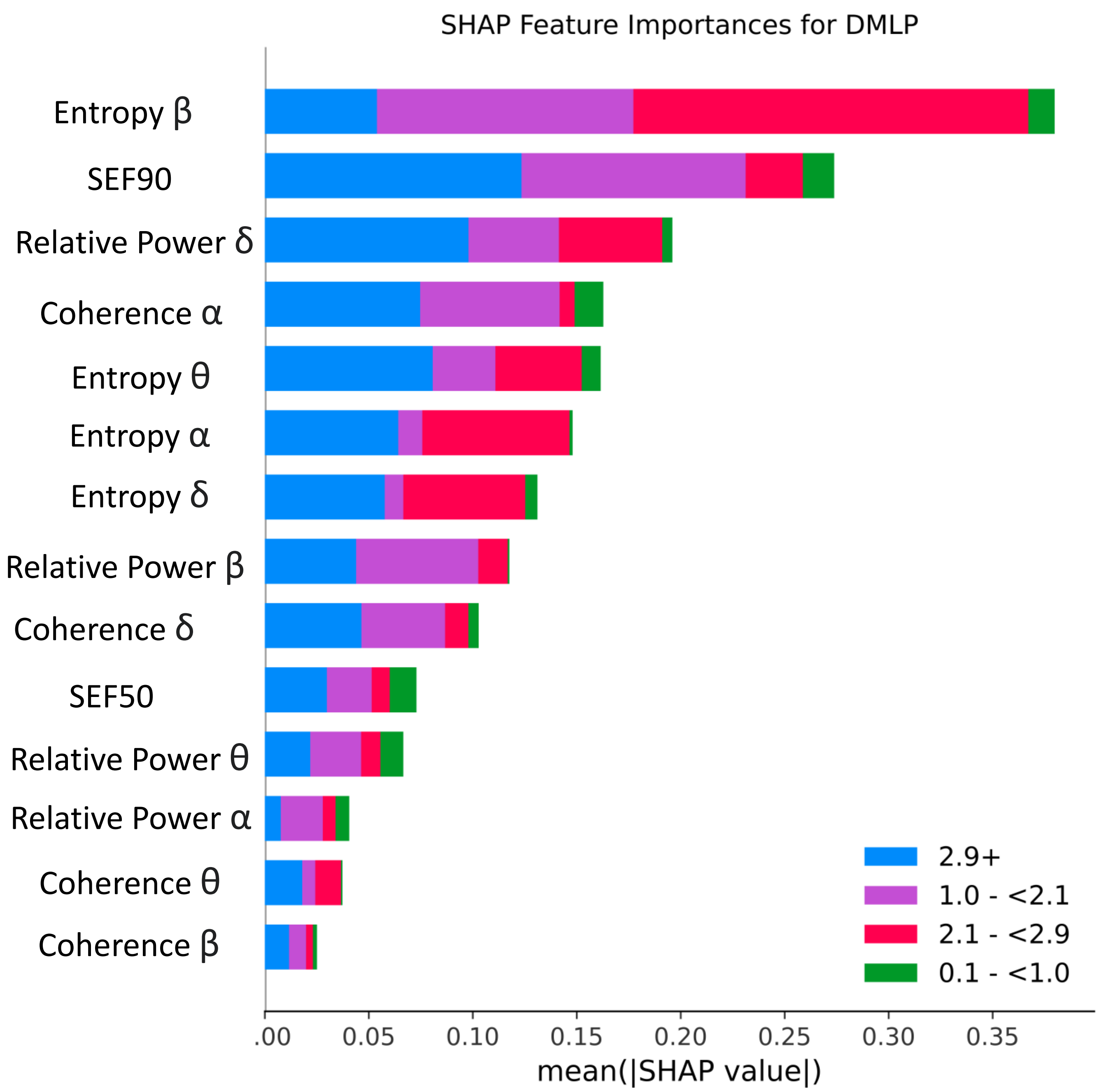
SHAP Feature Importances for DMLP
Entropy β
SEF90
Relative Power δ
Coherence α
Entropy θ
Entropy α
Entropy δ
Relative Power β
Coherence δ
SEF50
Relative Power θ
Relative Power α
Coherence θ
Coherence β
2.9+
1.0 - <2.1
2.1 - <2.9
0.1 - <1.0
.00
0.05
0.10
0.15
0.20
0.25
0.30
0.35
mean(|SHAP value|)

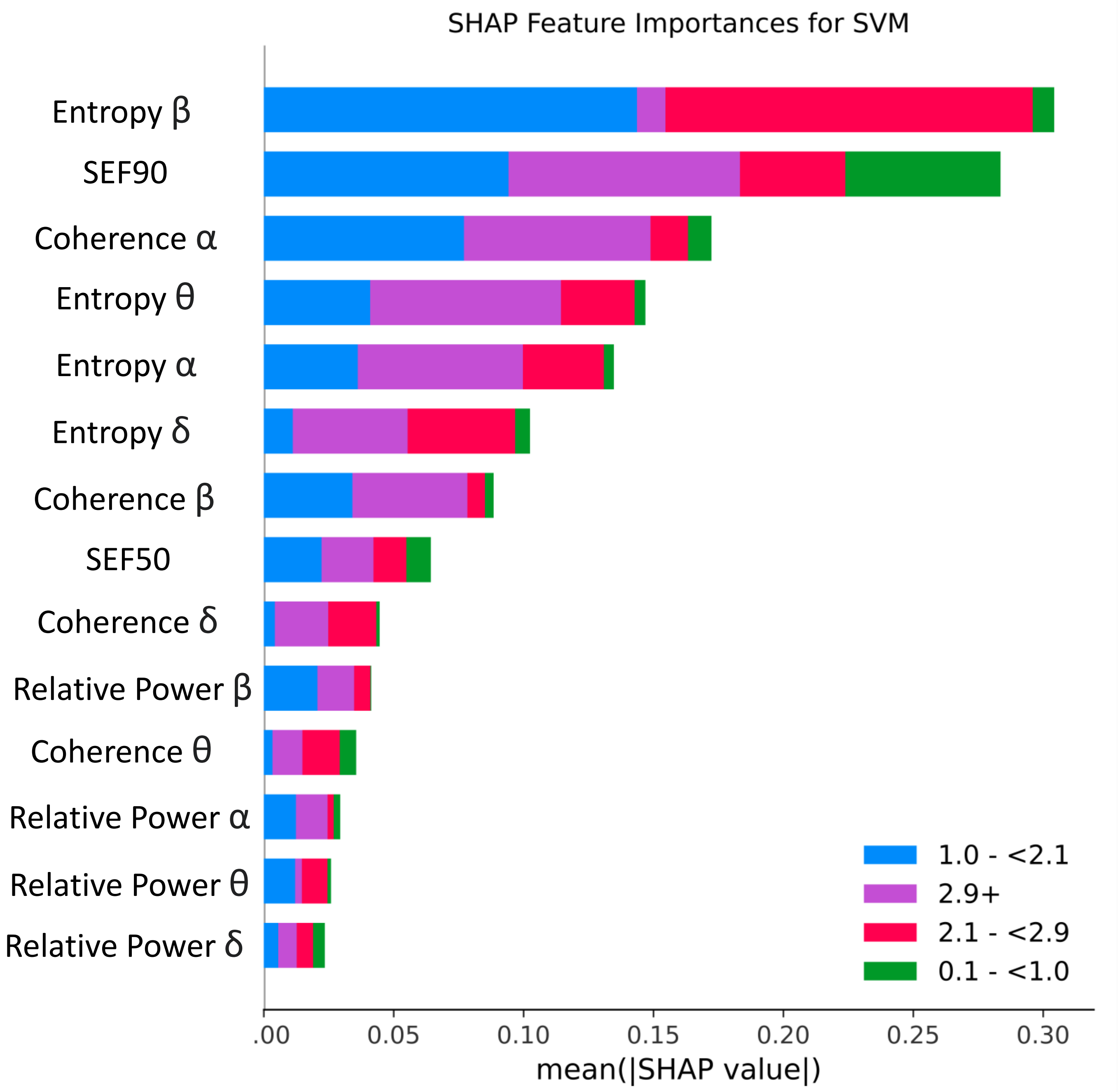

SHAP Feature Importances for SVM
Entropy β
SEF90
Coherence α
Entropy θ
Entropy α
Entropy δ
Coherence β
SEF50
Coherence δ
Relative Power β
Coherence θ
Relative Power α
Relative Power θ
Relative Power δ
1.0 - <2.1
2.9+
2.1 - <2.9
0.1 - <1.0
.00
0.05
0.10
0.15
0.20
0.25
0.30
mean(|SHAP value|)

Supplemental Figure 3: Without burst suppression ratio, ranked histogram of proportion (%) of each EEG features' contribution to the model. (top = KNN, middle = DMLP, bottom = SVM).

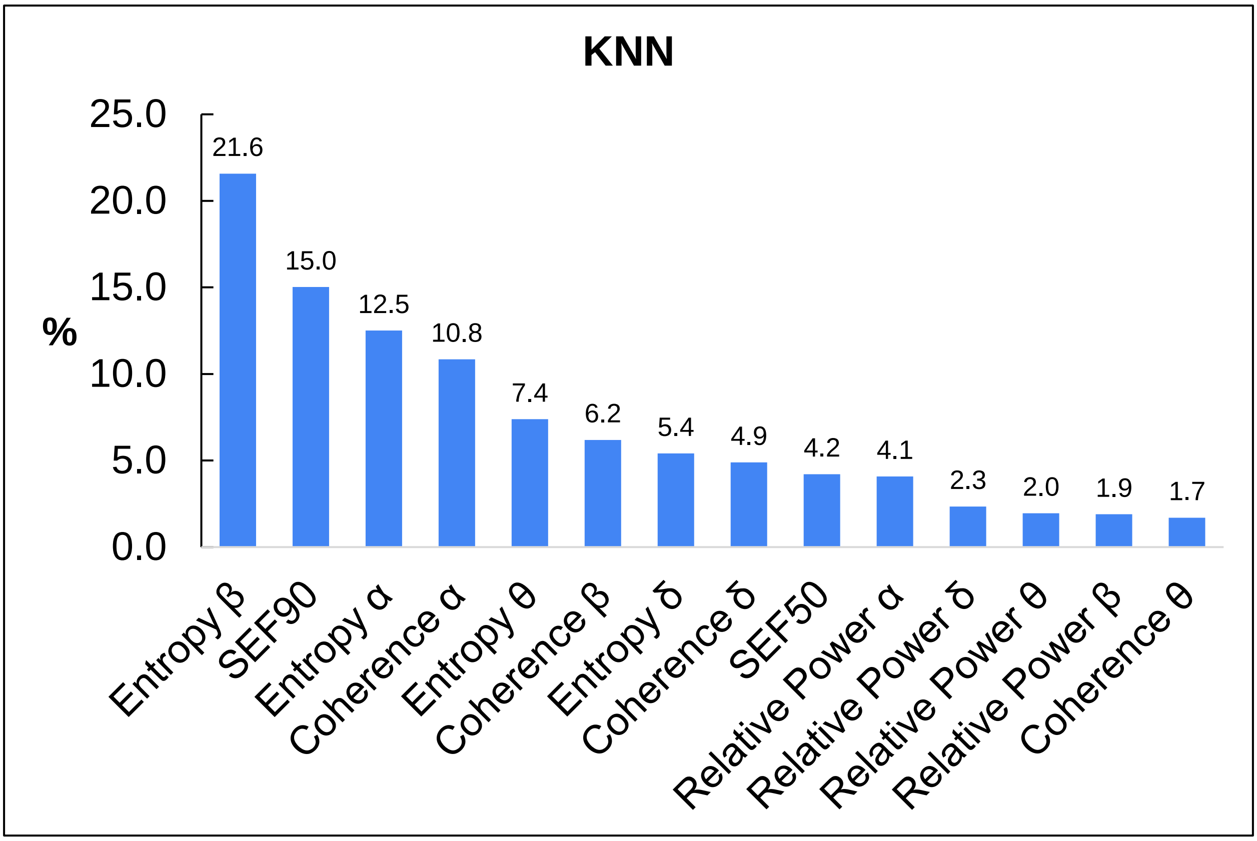

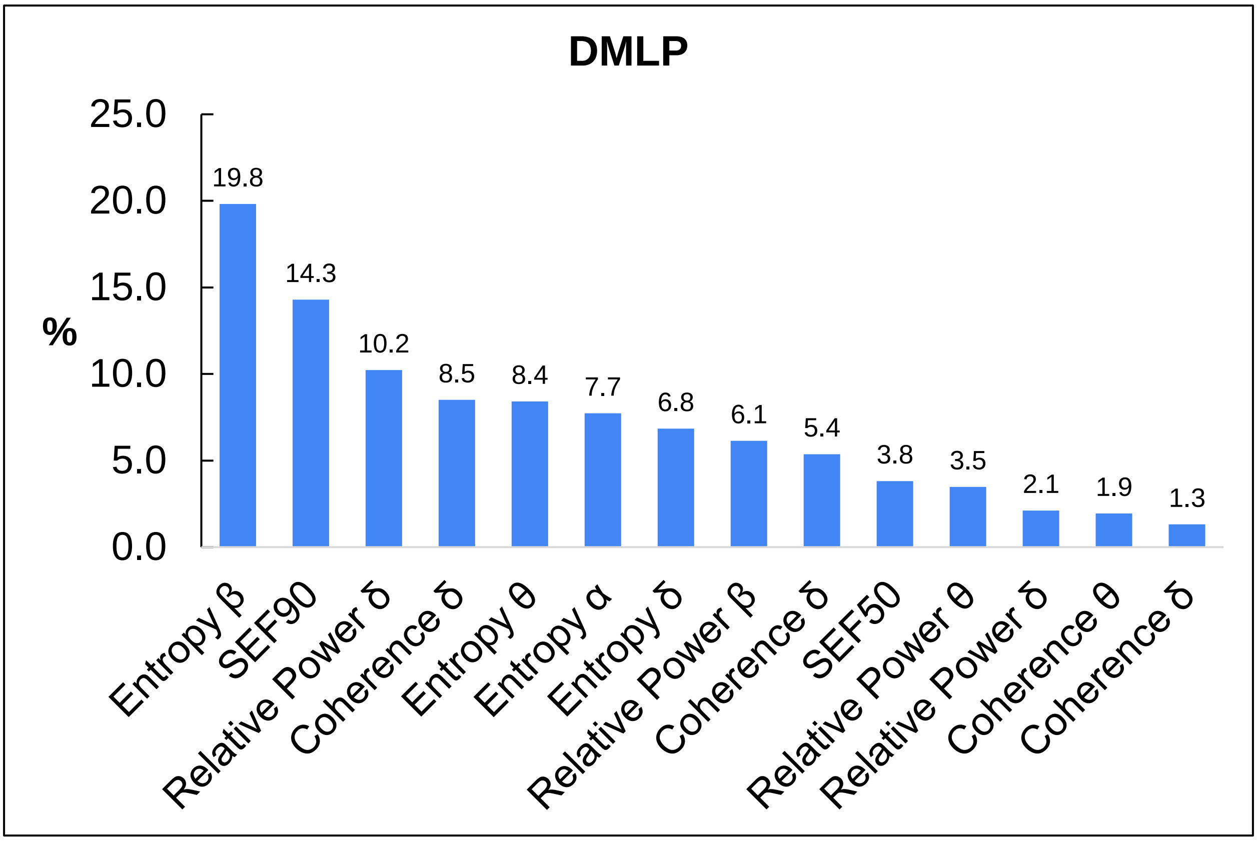
DMLP
%
25.0
20.0
15.0
10.0
5.0
0.0
19.8
14.3
10.2
8.5
8.4
7.7
6.8
6.1
5.4
3.8
3.5
2.1
1.9
1.3
Entropy β
SEF90
Relative Power δ
Coherence δ
Entropy θ
Entropy α
Entropy δ
Relative Power β
Coherence δ
SEF50
Relative Power θ
Relative Power δ
Coherence θ
Coherence δ

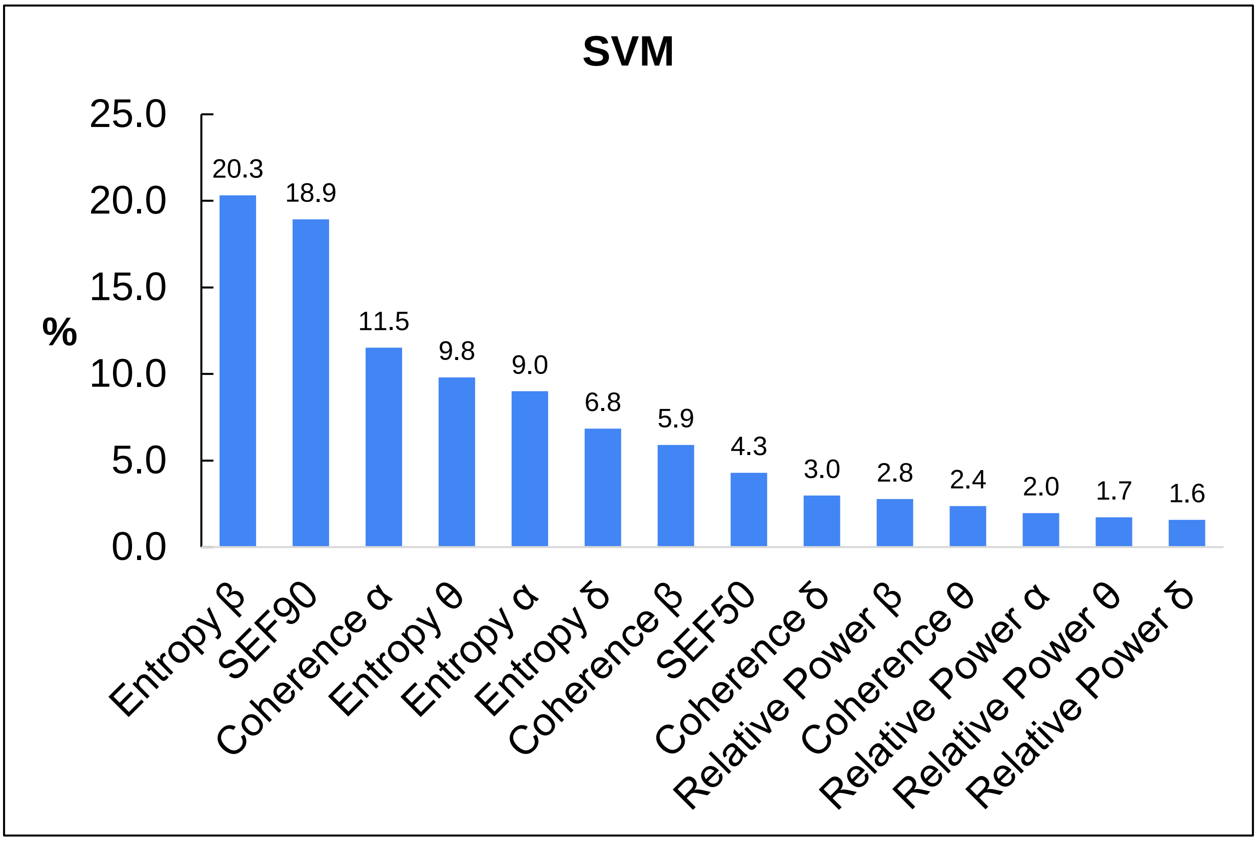
SVM
%
25.0
20.0
15.0
10.0
5.0
0.0
20.3
18.9
11.5
9.8
9.0
6.8
5.9
4.3
3.0
2.8
2.4
2.0
1.7
1.6
Entropy β
SEF90
Coherence α
Entropy θ
Entropy α
Entropy δ
Coherence β
SEF50
Coherence δ
Relative Power β
Coherence θ
Relative Power α
Relative Power θ
Relative Power δ
